# An Investigation of the Influence of Initial Roughness on the Friction and Wear Behavior of Ground Surfaces

**DOI:** 10.3390/ma11020237

**Published:** 2018-02-04

**Authors:** Guoxing Liang, Siegfried Schmauder, Ming Lyu, Yanling Schneider, Cheng Zhang, Yang Han

**Affiliations:** 1 Shanxi Precision Machining Key Laboratory, Taiyuan University of Technology, Yingze west street No. 79, Taiyuan 030024, China; lvming@tyut.edu.cn (M.L.); zclgx@126.com (C.Z.); hanyang0521@foxmail.com (Y.H.); 2 Institute for Materials Testing, Materials Science and Strength of Materials (IMWF) University of Stuttgart, Pfaffenwaldring 32, Stuttgart D-70569, Germany; siegfried.schmauder@imwf.uni-stuttgart.de (S.S.); yanling.schneider@imwf.uni-stuttgart.de (Y.S.)

**Keywords:** surface roughness, friction, wear, plastic deformation

## Abstract

Friction and wear tests were performed on AISI 1045 steel specimens with different initial roughness parameters, machined by a creep-feed dry grinding process, to study the friction and wear behavior on a pin-on-disc tester in dry sliding conditions. Average surface roughness (Ra), root mean square (Rq), skewness (Rsk) and kurtosis (Rku) were involved in order to analyse the influence of the friction and wear behavior. The observations reveal that a surface with initial roughness parameters of higher Ra, Rq and Rku will lead to a longer initial-steady transition period in the sliding tests. The plastic deformation mainly concentrates in the depth of 20–50 μm under the worn surface and the critical plastic deformation is generated on the rough surface. For surfaces with large Ra, Rq, low Rsk and high Rku values, it is easy to lose the C element in, the reciprocating extrusion.

## 1. Introduction

AISI 1045 steel groove components, with a depth to width ratio greater than two and a width of no more than 4 mm, are widely applied in small and miniaturized vane pump rotors [1]. Friction and wear usually occur in the contact zone between the vane and side profiles of the groove, which results in the failure of parts and extremely limits the performance of the pump. Higher friction coefficients means more pumping energy needed and higher operating costs. For most surfaces involved in the relative motion of contact pairs, the contact area of a rough surface is much lower than that of a smooth one. However, the contact pairs with rough surfaces usually lead to the speeding up of the damage process due to the friction and wear [2]. Hence, it is important to weaken the adverse impacts caused by the friction and wear. To do so, there are two common means, i.e., improving the surface integrity, especially decreasing the initial roughness value, and adopting special materials with excellent wear resistance. Between these two methods, the former is preferred thanks to its applicability for diverse machining processes and its lower costs [3,4,5].

Concerning the friction coefficient of AISI 1045 steel, Rech et al. [6] set up a friction model and described the friction coefficient at the interface of dry cutting of AISI 1045 steel with TiN coated carbide tools. The results showed that the friction coefficient has a strong dependence on the sliding velocity. Applying AISI 1045 steel, Iqbal et al. [7] studied the evolution of flow stress and the interface friction distribution. It was subsequently found that pure sliding is often an appropriate friction scheme for conventional machining at higher cutting speeds. B. Abdelali et al. [8] investigated the friction coefficient at the tool–chip–workpiece interface during the dry cutting of AISI 1045 and found that the sliding velocity is the most influential parameter compared to the contact pressure. In researching the friction and wear characteristics of AISI 1045 [9], it was found that the wear rate increases when the contact pressure and the sliding velocity increase. The wear mechanisms of AISI 1045 steel are mainly abrasion and adhesive wear. For the same material, Yang et al. [10] also studied the wear behavior and mechanisms using a pin-on-disk high wear apparatus. With increasing temperature, the microstructure of the worn surface was softened while the wear resistance rapidly decreased. Previous studies by Kubiak [11] explained the influence of surface roughness on the contact interface and evaluated the surface finishing as a factor in the friction and wear damage process. Wang et al. [12] studied the material formation on a worn surface layer during dry rubbing and found that the sliding friction triggers the shear deformation and heat in the contact zone. Hughes et al. [13] found a dislocation substructure and a mechanically mixed layer at different depths from the surface in sliding copper tests. Hughes et al. [14] also found that a structural description based on individual dislocations or dislocations arranged in smaller groups (tangles and cell walls) is valid at low strains. Meanwhile, a gradual transition in the scaling behavior is observed in the transition from small to large strain behavior and quantitative measurements are emphasized in the paper [15]. In addition, the asperity deformation model can explain the variation of friction coefficients and the stress concentration induced by friction. Two different patterns in a deformed surface imply the localized material flows. Lu et al. [16] investigated the plastic deformation on the surface by means of a surface mechanical attrition treatment and found that as more dislocations are activated, the grain size decreases gradually near the surface [17]. 

Other publications reported similar deformations caused by plastic flow in the surface of steels, such as the ball impact test on plastic steels [18], and steel cutting operations [19,20,21]. It was also found that the surface texture influences the plastic deformation in ductile materials after sliding [22]. M. Sedlacek et al. [23] investigated the correlation between surface roughness parameters and friction and found that the surfaces with higher kurtosis and negative skewness values tend to reduce friction. Meanwhile, they [24] investigated the influence of surface preparation on roughness parameters and the correlation between roughness parameters and friction and wear. A. Dzierwa [25] found that the initial surface topography has a significant influence on friction and wear levels under dry sliding conditions. W. L. Lu et al. [26] implied that the friction torque varies nonlinearly with the increase of surface roughness and surface roughness shows the opposite influence on wear under two different texture directions. Moreover, different topographies can also affect the tribological behavior due to the various contact mechanics. In studies of contact mechanics [27,28,29], the contact area rose linearly with the applied load and was inversely proportional to the RMS (root-mean-square) gradient of the surface profile. B. N. J. Persson et al. derived the boundary conditions for the stress probability distribution function for elastic, elastoplastic and adhesive contact between solids. Meanwhile, they studied the distribution of interfacial separation in the contact region between two elastic solids with randomly rough surfaces [30,31]. Hyun et al. [32] provided a method for determining contact geometry as a function of load on a constructed surface. 

Most previous studies emphasized the appearance of the worn grooves, the ridged features, the material delamination and wear after sliding tests [33,34,35]. These focused on the origins of the occurrence other than the material plastic deformation in the wear layer. Bumps, tears and crack-like features had also been observed in sliding tests [36]. The present observations also showed that wear particles can be developed during sliding in even only 1–2 sliding passes according to the folding-type mechanism. It suggested a mechanism for the initial steady wear that does not require chip formation by cutting due to the interaction of asperity surfaces [37,38]. 

Different initial roughness parameters result in different wear behavior, the important factors of which also include the initial steady wear transition time, the wear loss and the deformation due to shear strain in the subsurface. Until now, these influencing aspects seem to be neglected in revealing the wear behavior. 

The major proposition of the current work is to investigate the friction and wear behavior of AISI 10415 steel with different initial roughness parameters. Average surface roughness (Ra), root mean square (Rq), skewness (Rsk) and kurtosis (Rku) were used to characterize the surface roughness. One emphasis of our work is to explain variations in wear behavior owing to the above-mentioned factors.

## 2. Experiments 

### 2.1. Material 

**Figure 1:**
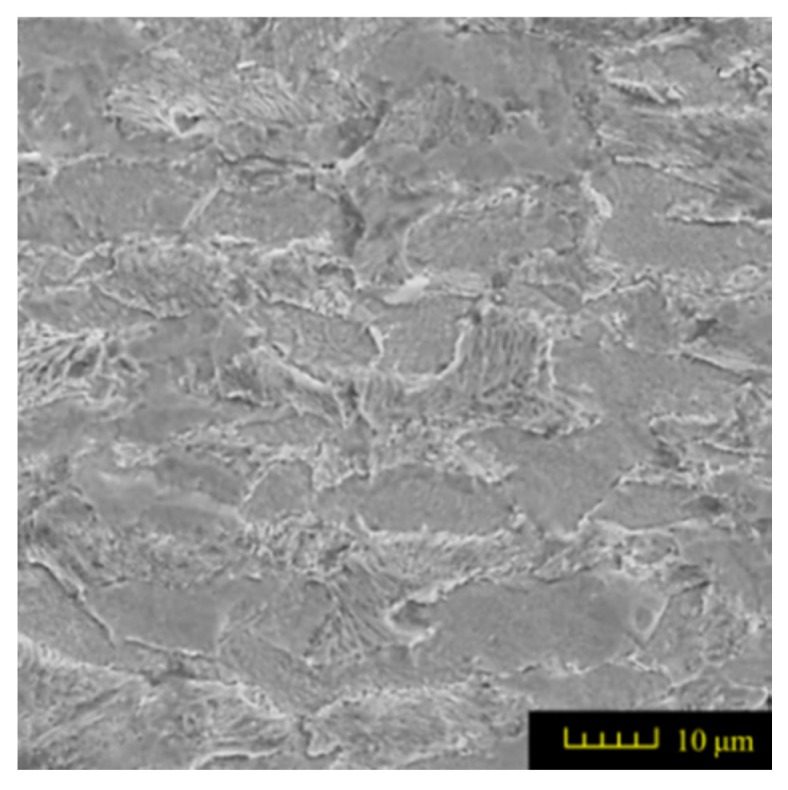
Microstructure of the investigated of AISI 1045 steel (etched with HNO_3_ 2.5%).

Commercially available AISI 1045 steel (HRC 34, Taiyuan Iron and Steel (GROUP) Co., Ltd., Taiyuan, China) was machined to six workpieces with the dimensions of 100 mm × 10 mm × 20 mm. The grinding process induced 25 mm deep grooves in each specimen. Each groove was formed in one grinding travel. The chemical composition of AISI 1045 steel is given in Table 1. The initial metallographic microstructures of the workpiece material before machining was observed by scanning electron microscope (SEM) and the results are shown in Figure 1.

It can be seen that AISI 1045 steel is composed of Ferrite–Pearlite and lamellar Pearlite with a proximal ratio of 1:1. The shape of ferritic and Pearlite grains is similar to polygonal and equiaxed grains. The size of Ferritic grains range from 20 to 30 μm mixed with same size pearlitic grains approximately. All features show that the workpiece is suitable for grinding. 

### 2.2. Grinding-Induced Variety of Surface Roughness

**Figure 2:**
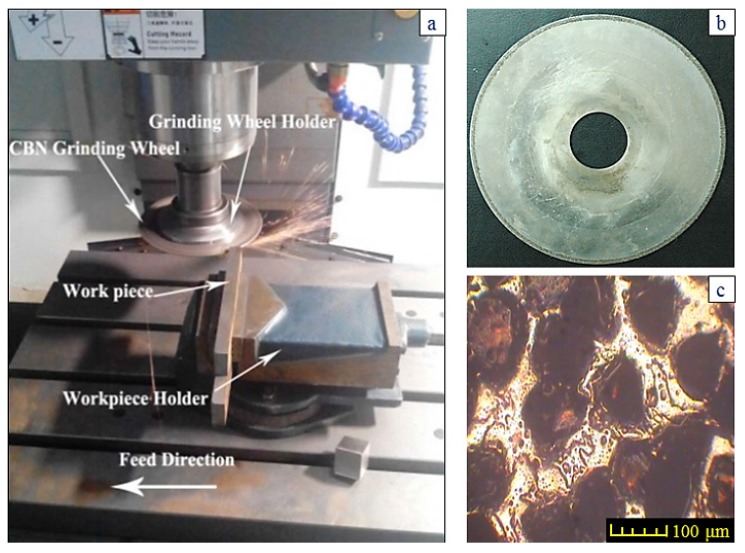
The detailed information of experiment setup: (**a**) The experiment setup; (**b**) the electroplated monolayer CBN; (**c**) CBN abrasive distribution.

The sample preparation is formed by the creep-feed dry grinding process (CFDG) on a MV-40 computer numerical control (CNC) precision vertical machining center (Wintec Precision Machinery Co., LTD.,Taiwan, China) and a spindle revolution between 10 and 10,000 r/min. The variety of surface roughness was induced by using different grinding parameters in the CFDG. Thermal damage is not allowed on the ground surface. The grinding setup is shown in Figure 2a. The grinding wheel used in the whole experiment was an electroplated monolayer CBN (Cubic Boron Nitride) with a diameter of 220 mm and a thickness of 2.0 mm, as shown in Figure 2b. The bond agent is Nickel–Fe alloy. The CBN abrasive with a grain size of 100–120 meshes was employed and its distribution on the wheel is shown in Figure 2c. 

The detection instruments used for the micrograph are the TESCAN scanning electron microscope CSM-100X (SEM) (TESCAN CHINA, Ltd., Shanghai, China), M-800X optical microscope (Shanghai Institute of Optics and Fine Mechanics. CAS, Shanghai, China) and Think Focus SM-1000 3D scanning system (Sixian Photoelectric technology (Shannghai) Co., Ltd. Shanghai, China). Oxford energy dispersive spectrometer X-act (EDS) (Oxford Instruments, Abingdon, UK) was applied to measure the weight percentages of elements.

**Figure 3:**
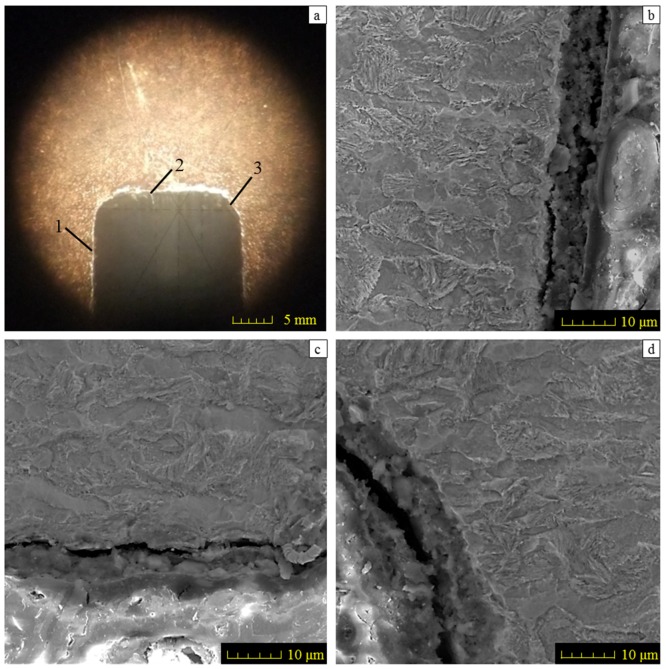
Microphotography and subsurface metallographic texture of ground specimen: (**a**) Microphotograph of specimen; (**b**–**d**) subsurface metallographic texture on groove side-surface, on groove bottom and in groove transition fillet area, respectively.

The design requirements must be fulfilled for the groove geometry, i.e., the width of 2 mm, the depth of 25 mm and the fillet radius no more than 0.3 mm. The feature of the microstructure on the ground surface should not be changed in CFDG. Figure 3a shows the amplified photographic structure of the groove cross-section near to the sample bottom. Three positions of the groove are presented. Position 1 denotes the groove side, position 2 denotes the groove bottom and position 3 denotes the groove fillet. After the grinding process, Figure 3b–d show the corresponding metallographic microstructures at three different positions. The common characteristic of these microstructures lies in the consistency of the ferrite and the pearlite. A comparison of the microstructures before and after the CFDG, the subsurface textures showed in Figure 3b–d present good agreement with that of Figure 1. Furthermore, and as desired, no phase transformation appears during the grinding process. The results confirm that the CFDG does not introduce thermal damage into the subsurface of ground specimens. It may be concluded that the CFDG is a technique with the potential to improve the grinding performance during machining components with narrow–deep groove structures. 

Sectioning along the groove longitudinal direction, the side profiles of the ground surface were obtained for preparing the samples. The size of each sample is 25 mm × 10 mm × 10 mm. The samples with 3D topography, SEM microstructure and roughness parameters are listed in Table 2. The samples were conducted with various grinding feed rates (*v_w_*) when the grinding speed is equal to 93 m/s. Each type of roughness parameter listed in the Table 2 is an averaged value measured on five different locations.

From Table 2, it can be seen that the textures of the ground surface are quite different from each other, and that the initial roughness parameters are not obvious as the feed rate decreases. The feed rate has observable effects on the surface texture and roughness values during CFDG.

**Figure 4:**
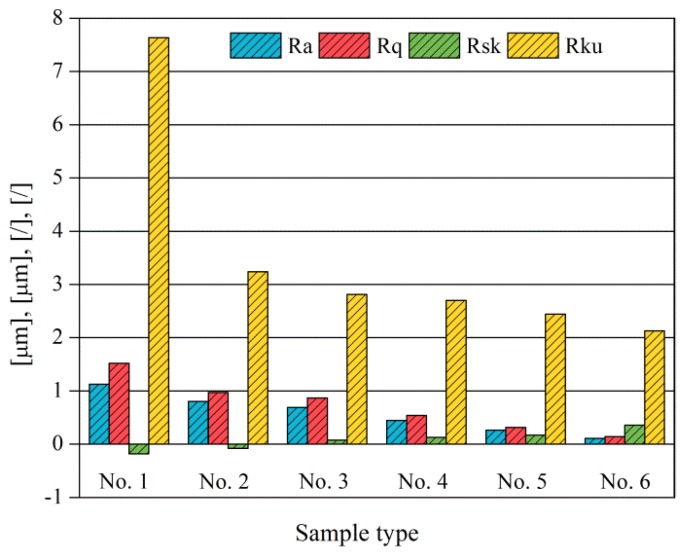
The surface roughness variation trend of different specimens.

It can also be seen that similar surfaces have very different standard roughness parameters, as shown in Table 2. The parameter of Ra describes the height variations. The parameter of Rq has a higher sensitivity to deviations from the main line than Ra. The parameter of Rsk can be used to describe occasional deep valleys or high peaks. The parameter of Rku describes the probability density sharpness of the profile [24]. The roughness parameter results of Ra, Rq, Rk and Rsk are collected in Figure 4.

### 2.3. Friction and Wear Testing 

**Figure 5:**
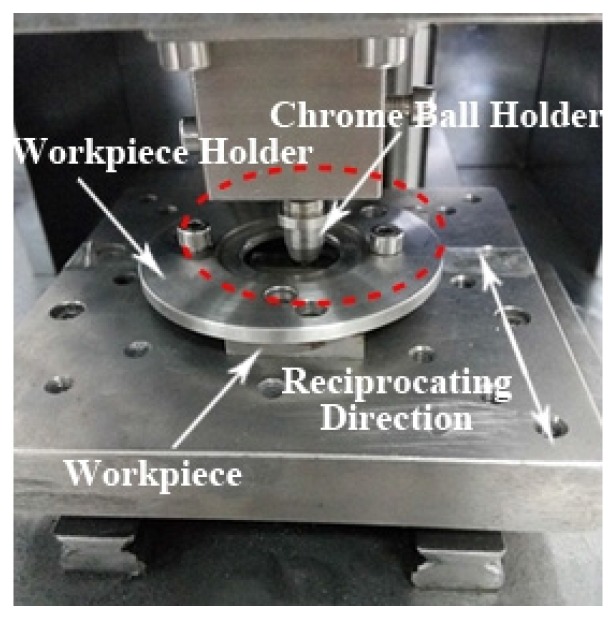
The pin-on-disc tester.

Before the friction and wear test, the GGr15 and AISI 1045 steel specimens were cleaned in an ultrasonic cleaning machine using an acetone bath. Then the specimens presenting on the ground surface were employed for the friction and wear testing on the CFT-1 (Lanzhou Zhongke Kaihua Technology Development Co., Lanzhou, China), multi-function, pin-on-disc machine, as shown in Figure 5. During the experiment, the temperature of the experiment was 20 °C and the relative humidity was 23–25%. The cylindrical pin contains a 5 mm diameter ball made from the GCr15 bearing steel with a hardness of 63 (HRC) and the chemical compositions are listed in Table 3. 

The nylon cage was utilized to hold the ball and avoid the vibration and noise. An external load of 10 N was applied along the axis direction of the cylinder pressing on the aforementioned ball in the sliding test. The stroke of the table was set as 5 mm and the spindle rotation was 800 r/min. Meanwhile, the friction frequency and amplitude were recorded by an acceleration sensor (JHK-ZHJ-3) (Beijing Centrwin Technology. Co., Ltd. Beijing, China.) together with the data acquisition instrument (RP-3500) (Rigol Technologies INC, Suzhou, China). The samples with various surface roughness parameters were tested to obtain the friction coefficient, the friction frequency, the friction amplitude, the wear width, the wear depth, the weight and the volume loss, the friction coefficient and then to observe the material deformation in the subsurface. 

## 3. Results and Discussion

### 3.1. Friction Coefficient

**Figure 6:**
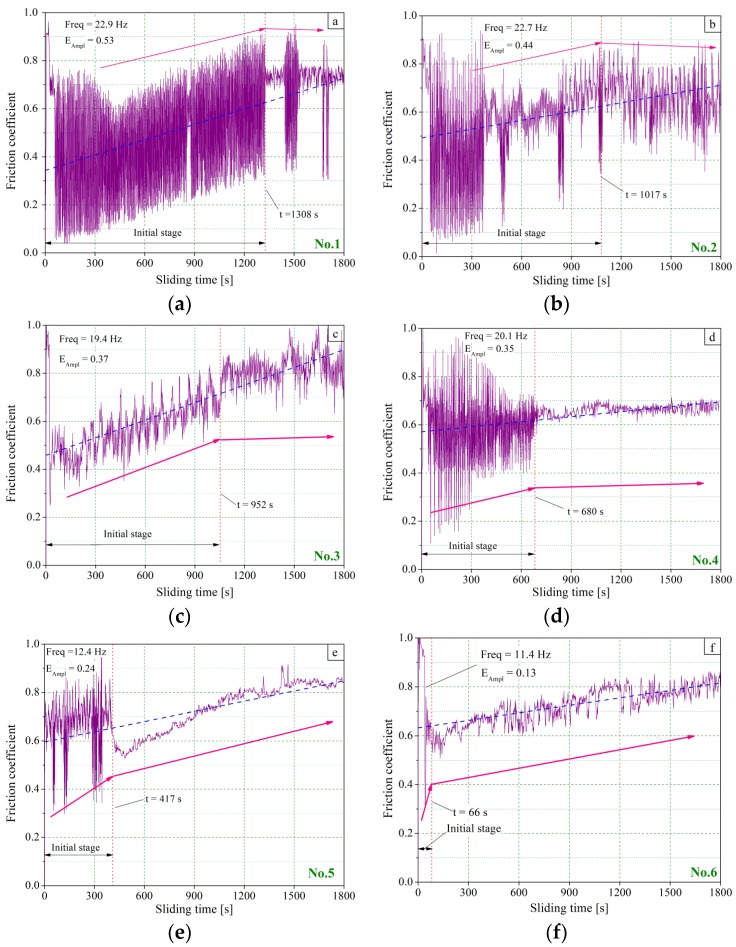
Time dependencies of the friction coefficients: (**a**–**f**) No. 1–No. 6 coefficient as a function of the sliding time.

The evolution of the friction coefficient versus time was obtained for a running time of 1800 s, as shown in Figure 6. The initial stage wear transition time, the friction coefficient oscillation frequency (Freq) and its mean amplitude (E_Ampl_) value are also noted in the figures. 

Figure 6 shows the evolution of the friction coefficients of the each sample as a function of the sliding time. One can conclude that the measured curves are characterized by the oscillations of the friction coefficients regardless of the values of amplitudes and frequencies. A similar phenomenon has been presented in the literature [39]. The vertical red dot line presents the start point of the stable friction time. As a criterion of distinguishing the initial wear from the steady wear, there is a flat tendency with a sudden reduction in amplitude occurring on the friction coefficient curves and the transition of the initial steady stage is identifiable. The friction coefficient linear fitting is also marked with a blue dashed line and a pink arrow line to denote the tendency of the friction coefficient in different stages. 

For sample No. 1, the friction coefficient fluctuates greatly (Figure 6a). The average friction coefficient is 0.76. The value of E_Ampl_ is 0.53 and Freq is 22.9. In the initial stage, the friction coefficient experiences a relatively steep rise. After the time of 1308 s, it shows a slightly declined tendency and smooth pattern. 

A similar pattern occurs with sample No. 2, with a friction coefficient with a drastic fluctuation in the initial stage, and almost equivalent values of average friction coefficient (0.72), E_Ampl_ (0.44) and Freq (22.7). The clear distinction information is the transition time of 1017 s (Figure 6b). 

In Figure 6c, it can be observed that the fluctuation mode of the friction coefficient is different from the curves displayed in Figure 6a,b. The friction coefficient performs similar fluctuation modes during the sliding test. It does not seem to burst the sudden reduction of the amplitude in Figure 6c. According to the results monitored by the data acquisition instrument, the frequency of the friction coefficient decreased rapidly at the time of 952 s, and then became flat. The values of the friction coefficient (0.65), E_Ampl_ (0.37) and Freq (19.4) can also be obtained in the sliding test.

In Figure 6d, greater fluctuation of the friction coefficient is also distributed in the initial stage. The distinction of amplitude is much evident. It is obvious that the curve becomes smooth behind the transition time of 680 s. The values of the friction coefficient (0.63), E_Ampl_ (0.35) and Freq (20.1) were found in the test.

Comparing the results in the aforementioned four figures, a prominent sudden tendency of friction coefficients occurred at time of 417 s (Figure 6e), and 66 s (Figure 6f). Analogously, the values of friction coefficient (0.57), E_Ampl_ (0.24) and Freq (12.4) were found in sliding sample No. 5. The values of friction coefficient (0.53), E_Ampl_ (0.13) and Freq (11.4) were found in sliding sample No. 6. 

The results of the curves show that the transition time at the end of the initial stages is different from each sample in the sliding test.The time at that point was marked with the red dot line in each graphy. After this point, the trends of the friction coefficient remains relatively steady and flat, as shown by the pink arrow lines marked in Figure 6. According to the fitting line, the friction coefficient of all samples has a slightly increasing tendency, although the testing results are very unstable at the initial stage. After the transition time, the gross trend of the curve becomes flat and tends to be smooth, which indicates that the transition from the initial wear to the steady wear takes place. Furthermore, wave modes of the friction coefficient present diversity in frequency and oscillation amplitude as well as the transition time of the initial stage.

According to the description mentioned above, the largest E_Ampl_ of 0.53 and the largest Freq of 22.9 Hz were monitored in sliding sample No. 1. On the contrary, the smallest E_Ampl_ of 0.13 and the smallest Freq of 11.4 Hz were detected in testing sample No. 6 (Figure 6f). Overall, the E_Ampl_ and the Freq show a declining tendency, with a decrease of the initial roughness values. This performance, appearing in pin-on-disk testing, has been declared in other publications [40]. Besides, a similar decline also happens in the initial steady transition time in the sliding test.

**Figure 7:**
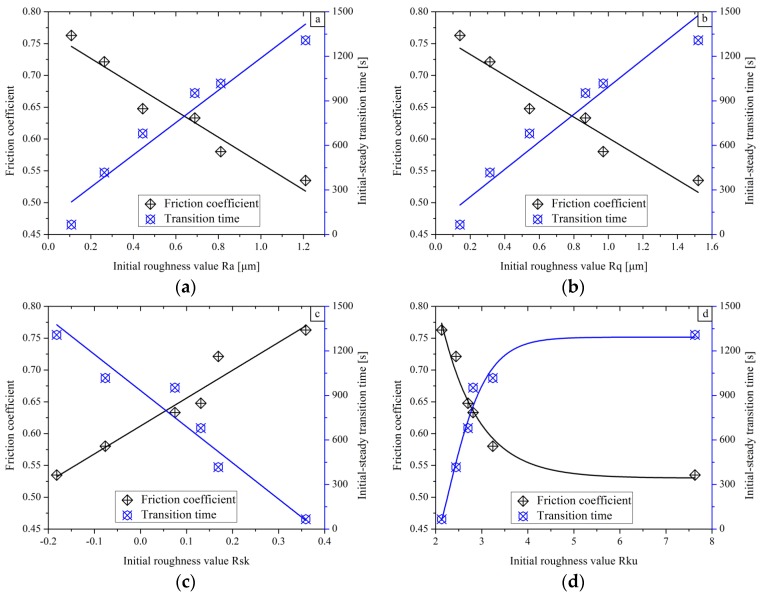
Variations of friction coefficient and initial-steady transition time with respect to initial roughness parameters: (**a**–**d**) corresponding to Ra, Rq, Rsk and Rku, respectively.

Focusing on the complete results shown in Figure 6, the initial roughness parameters play a crucial role in determining the friction coefficient and the initial steady transition time directly, while their gross tendencies are shown in Figure 7. 

In Figure 7a,b, the averaged friction coefficient decreases with the increasing of the initial roughness values of Ra and Rq, while the initial steady wear transition time behaves in the opposite manner. The lower the friction coefficient is, the longer the transition period will be. Under this condition, for a surface with high roughness values of Ra and Rq, the friction and the wear behavior will reach their own stable stage more quickly and remain at the flattened level until the end of the test. Hence, the wear transition mode is easy to control in the running components. Analogously, smaller initial roughness values of Ra and Rq lead to a higher friction coefficient, which is in agreement with the results reported by other authors [41]. In the case of samples with larger initial roughness values of Ra and Rq, a longer period would be taken to form the new surface in the sliding track. Consequently, specimens with an asperity surface need much more sliding time to reach the stable stage. The wear transition will come into the stable stage when the initial rough surface is removed thoroughly.

In Figure 7c, the initial roughness value of Rsk also has an effect on the average coefficients. With the value of Rsk increment, the friction coefficient increases and the initial steady wear transition time shortens. The parameter of Rsk with a negative value means that deep valleys and few high peak are generated on the sample in the CFDG process, which looks like a plateau smooth surface. Therefore, a small tangential force will be induced during sliding over each valley and a smaller friction coefficient is found on the rough surface in sliding tests, such as for sample No. 1 and No. 2. Besides this, the new surface in the sliding track will be generated within a short time.

In Figure 7d, it can be seen that the averaged friction coefficient first increases and then becomes flat with a rising Rku, and the initial steady wear transition time tendency introduces the opposite case. The surface has some high peaks and deep valleys when the value of Rku exceeds three. Negative Rsk and Rku exceeding three occurred in samples No. 1 and No. 2, in which deep valleys and high peaks formed in the CFDG. One can also find that samples with larger Rku result in smaller average friction coefficients. The larger the Rku, the higher the cantilever between the valley and peak will be. Then the strength of the cantilever decreases. Consequently, the tangential force generated in the sliding test declines as well as the friction coefficient. Nevertheless, more sliding travel can realize new surface.

**Figure 8:**
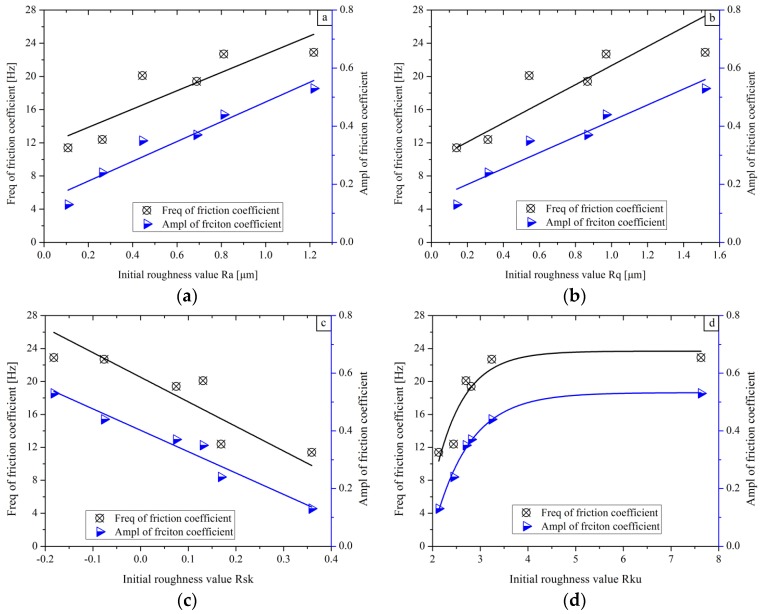
The friction parameters of Freq and EAmpl as a function of the initial roughness parameters: (**a**–**d**) corresponding to Ra, Rq, Rsk and Rku, respectively.

The results noted in Figure 6 also show that the initial roughness parameters also affect the oscillation Freq and the oscillation E_Ampl_, as given in Figure 8. 

In Figure 8a,b, it can be seen that a larger roughness of Ra and Rq lead to a higher frequency and amplitude of the friction coefficient. Fitting lines indicate that the Freq and E_Ampl_ of friction coefficients present an incremental tendency when the surface becomes rougher. If the texture along the horizontal surface is severely uneven, the oscillation Freq will become high. If the texture perpendicular to the surface is intensely bumpy, the oscillation E_Ampl_ will be greater. Therefore, the rougher surface will induce a larger fluctuation for the tangential forces along the sliding direction. When the ball slides over the surface, the friction coefficient curve will present various vibration modes and the friction coefficient is not stable on the rough surface. Furthermore, it is reported in [42] that diverse surface textures could be a reason inducing the oscillation in sliding tests.

In Figure 8c, the Freq and the E_Ampl_ of the friction coefficient present the decline tendency as the Rsk increases. A larger positive Rsk means a sample surface with high peaks and shallow valleys. It is reported in publication [23] that positive Rsk can provide a greater actual contact area and a large number of peaks between sliding ball and surface. As a result, the smaller Freq and E_Ampl_ of the friction coefficient can be noted on the samples with large Rsk in sliding tests. 

On the contrary, it can be seen in Figure 8d that both Freq and E_Ampl_ of the friction coefficient first increases and then becomes flat with the increase in Rku. The sample with the large Rku results in a high Freq and E_Ampl_ friction coefficient due to a lot of high peaks and deep valleys on the sample surface. 

### 3.2. The Cross-Section Profile of Worn Surface Analysis

**Figure 9:**
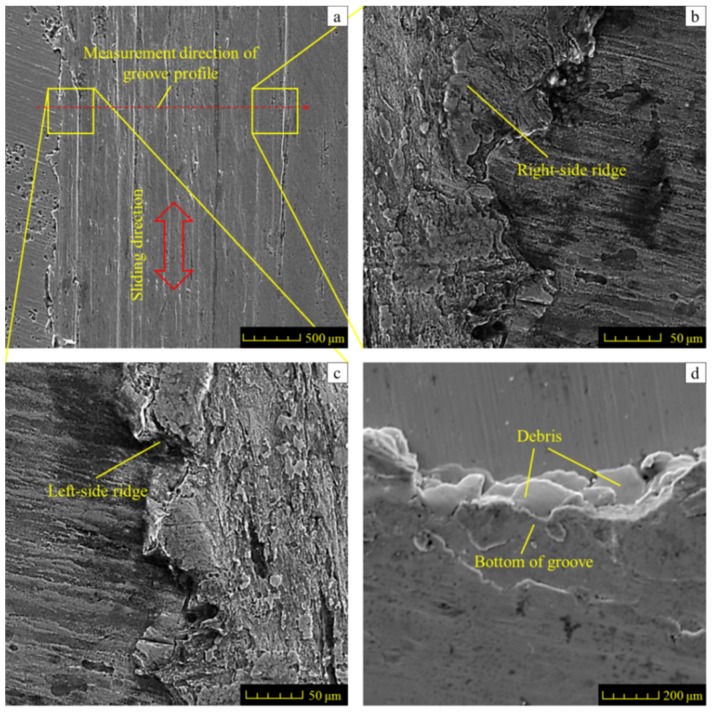
Microphotographs of the worn surface: (**a**) microphotograph of the worn groove surface; (**b**,**c**) microphotographs of the left-side and right-side ridge of the worn groove respectively; (**d**) section profile of the worn groove.

To understand well the friction and wear behavior of AISI 1045 steel, the worn grooves and their cross-section and wear loss were studied after the sliding test. The representative worn surface topography is shown in Figure 9. 

**Figure 10:**
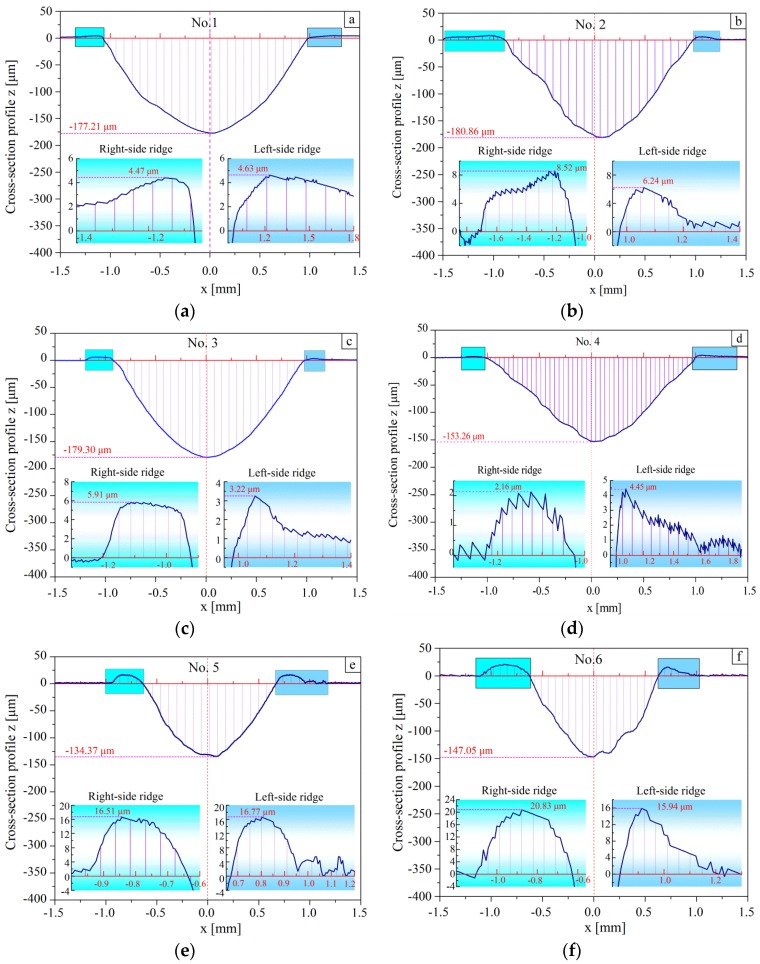
Cross-section profiles of worn grooves for mentioned six specimens: (**a**–**f**) cross-section profile, the ridges on both sides of the worn grooves in the middle of the sliding track.

An enlarged view of the worn groove is shown in Figure 9a. The ground surface is worn out along the sliding direction and a typical wear mark with a width of about 2 mm can be clearly observed. On both sides of the worn grooves, two stripes of ridges were produced due to the plastic deformation in the sliding test, as indicated in Figure 9b,c. Such ridges could be attributed to the plastic flow. When the GCr15 ball slides against the surface, the displacement in the sliding direction causes the tangential force to induce the material plastic deformation which mainly flows in two directions, i.e., the sliding direction and the perpendicular direction to the sliding track. Along the sliding direction, the material was partly pulled up and bumped ahead of the ball, then was compressed in the groove or removed from the worn surface. Shown in Figure 9d, compressed bumps that are not removed are helpful for debris to grow in the reciprocal sliding interaction. The component force perpendicular to the sliding track is caused by the normal force, which compresses the material flow to two sides of the groove, then the ridges formed. Two-dimensional cross-sectional profiles of the groove are obtained in the middle of the track, as shown in Figure 10, and the shapes of the ridges on both sides of the worn groove are also presented. In each graph, the depth of the worn groove and the height of the ridges were measured.

For the cross-section profile of sample No. 1 (Figure 10a), the groove has a profile with dimensions of 177.21 μm depth and 1.92 μm width. On both sides of the groove, two ridges can be observed. The height of the left-side ridge is 4.47 μm and the right-side ridge is 4.63 μm. For the profile of sample No. 2 (Figure 10b), the groove with a depth of 153.6 μm and a width of 2.08 μm appears shallower than that of sample No. 1. The height of left-side ridge is 8.52 μm and the right-side ridge is 6.64 μm. For sample No. 3 (Figure 10c), the groove profile shape has a similar geometry as aforementioned. A profile with a depth of 179.30 μm and a width of 1.64 μm was obtained. No. 4 (Figure 10d) indicates a groove profile of 153.26 μm depth and 2.02 μm width. The two ridges are of 2.16 μm and 4.45 μm depth. In Figure 10d, the ridge height (left-side, 16.51 μm and right-side, 16.77 μm) on both sides of the groove increases. The groove profile presents 134.37 μm depth and 1.31 μm width (Figure 10e). As for the sample No. 6 (Figure 10f), the depth of the worn groove is 147.05 μm. The ridge height (left-side, 20.83 μm and right-side, 15.94 μm) and groove width (1.38 μm) can also be measured after the sliding test.

Overall, the depth of the grooves varies with respect to the initial roughness value and there is an ascending trend of depth with the increment of the initial roughness value. It may be possible to conclude that the larger initial surface roughness value of the specimens results in a longer running-in duration [43]. Furthermore, the longer running-in duration causes the longer initial wear stage. In this stage, the rough surface may lead to rapid damage by the sliding ball in the contact zone and a large amount asperity material has been flattened or removed. Hence, it is reasonable to conclude that the depth of the groove increases as the initial surface roughness values of Ra and Rq become larger. The cross-section profiles also show that the plastic deformation occurred on the sides of the worn grooves. Furthermore, there is no obvious regular change in the height of the ridges when the initial roughness value varies.

Another interesting phenomenon is that the ridges are almost very rough. For sample No. 5 (Figure 10e) and sample No. 6 (Figure 10f), higher ridges occurred in the samples with initial roughness parameters of smaller Ra, lower Rq, larger Rsk and smaller Rku. According to the topography of the samples listed in Table 2, a rougher surface has a higher rib-like uplift than a smoother surface. It looks like the ribs adhere to the steel plate. A rougher surface with larger Ra and Rq, especially with higher Rsk, means a larger height of the ribs, which increases the strength in a certain area close to the surface. When the ball is sliding over the surface, the plastic deformation perpendicular to the sliding track becomes more difficult than that along the sliding track. Then, little plastic deformation is produced perpendicular to the sliding track and high ridges can hardly be found in samples with a rough surface.

**Figure 11:**
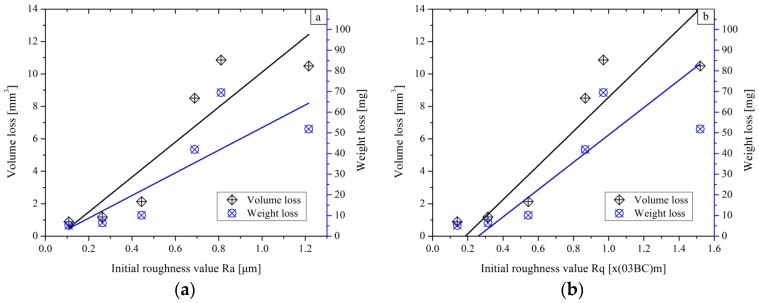

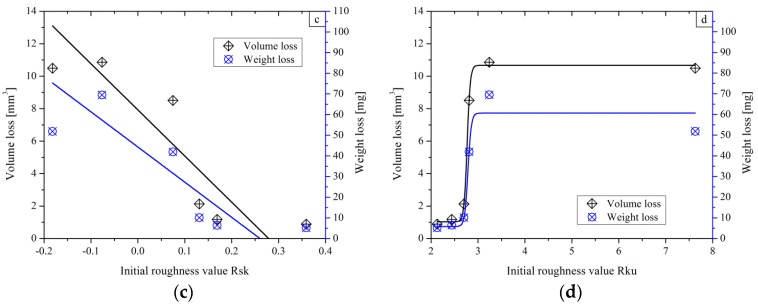
The volume and weight losses as a function of the initial roughness value: (**a**–**d**) corresponding to Ra, Rq, Rsk and Rku, respectively.

The samples were completely cleaned with anhydrous alcohol and weighted using a digital balance with an accuracy of ±0.1 mg. By comparison with the sample mass weighted before the wear tests, one obtains the weight loss. The results of the volume loss were derived by evaluating the mean area of three measured cross-sections and multiplying by the length of the wear track. These results also indicate a trend for the wear rates for the different surfaces. For a better understanding of the wear behaviour of AISI 1045 steel specimens with various roughness values, the weight/volume loss versus the initial roughness is plotted in Figure 11. 

In Figure 11, the scale of the volume loss is from 0 to 14 mm^3^ and the scale of the weight loss is from 0 to 109.9 mg. When one divides 109.9 by 14, a ratio of 7.85 mg·mm^−3^ (the density of specimen) is achieved. Normally, the slopes of two fitting lines should be consistent. It is obvious that the slope of the weight loss is smaller than that of the volume loss, which can be explained by the fact that the side ridge volume was not taken into consideration when calculating the volume loss. However, the material of the ridge on both sides of the groove comes from the material deformed due to the plastic deformation, as mentioned before. The trends of linear fitting lines increase with the increment of the initial roughness value Ra and Rq (Figure 11a,b). Inversely, the wear loss decreases with the increment of Rsk (Figure 11c). In the case of the dry sliding, the dominant wear mechanism is abrasion [23]. The peaks on the surface with a high Rku value will easily get worn away and become particles. The generated particles together with the sliding ball intensify the rubbing effect, which gives rise to larger wear loss generated on the sample with a high Rku value, as shown in Figure 11d.

In fact, surfaces with large roughness values of Ra and Rq, together with high Rku and more negative Rsk, imply more high peaks and deep valleys existing on the samples. In the initial stage, the size of the worn particles is generally between 10 and 100 μm. Those particles cannot get into the traps between the peaks and valleys on the surface and begin to cut the material as a tool driven by the sliding ball. Therefore more material in the contact zone will be removed. 

A smoother surface has a lower volume and weight loss than a rougher one. As for the friction and wear, a possible detail found in the experiment is that the loss is approximately on the same level when the initial roughness is below a certain limit (about Ra 0.3–0.5 µm, Rq 0.1–0.4 µm). These results indicate that decreasing the roughness values of Ra and Rq does not involve too much benefit in terms of improving the wear resistance when the surface roughness value is low enough.

### 3.3. Analysis of Material Plastic Deformation in the Worn Layer

**Figure 12:**
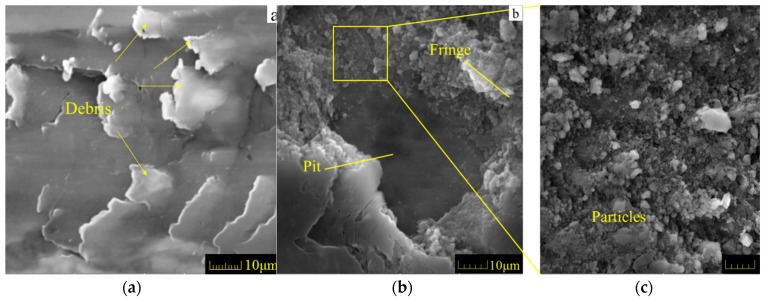
SEM graphs of worn surface: (**a**–**c**) the similar characteristics of fretting wear in localized zone.

Wear debris and particles have been observed on the worn surfaces after sliding the sample with a rough surface, as shown in Figure 12a. The wear debris may be explained by the fretting wear mode occurring in a localized zone [44]. When the ball slides over the ridges between two stripes on the rough surface, oscillation with a small amplitude appears only in a localized zone between the ball and the rough surface. Then, oscillatory tangential vibrations are generated in the relative sliding motion. Such a phenomenon seems to be caused by the fretting wear process. Fretting wear induces damage to pits in which powder particles are usually filled, as shown in Figure 12b,c. Furthermore, wear fringes, grooves as well as surface micro-cracks are produced in the localized zone.

**Figure 13:**
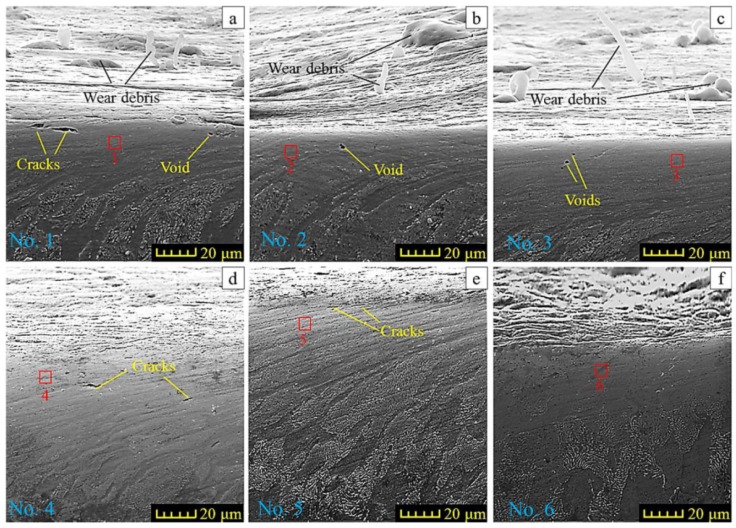
SEM graphs of plastic deformation under the worn surface: (**a**–**f**) present the plastic flow microstructures of No. 1–No. 6 specimens, the red rectangular area denotes the detection position of the EDS.

In order to obtain a more thorough understanding of the initial roughness effect on the wear behavior, SEM was used to trace the material plastic deformation. The specimens to be examined were sectioned perpendicular to the worn surface and along the sliding track. The features observed in the cross section of the worn samples are shown in Figure 13. 

For the samples No. 1–No. 4, some extreme plastic deformation was generated in the subsurface layer (Figure 13a–d) with a thickness ranging from 10–50 μm. Specimen No. 5 has a mild deformed layer with a thickness of about 20 μm, and little plastic deformation is observed in the subsurface layer of specimen No. 6. Structurally, this deformation layer may be divided into a fragmentation zone (close to the worn surface) and a textured grain zone (under the fragmentation zone). It is clear that a larger deformation is generated in the fragmentation zone. One can see that the strain gradually grows, starting from the deepest layers of the textured grain zone to the fragmentation zone. The deformation of the grain boundaries is too critical to be observed besides a few stripes of distorted residual boundaries remaining in the subsurface layer. It looks like a phenomenon of material flowing below the subsurface layer, although the material is not mobile in accordance with the rheology theory. In the textured grain zone, the grains are slightly deformed by the shear stress coming from the fragmentation zone. The critical deformation in the subsurface layer is presented in Figure 13a (sample No. 1). However, a slight deformation is observed in the sample No. 6 (Figure 13f). This can be explained by the fact that the initial roughness parameters with higher Ra, Rq and larger Rku induce larger tangential forces along the sliding track when the normal force is constant. The larger tangential force causes the greater shear stress and deformation generated in the subsurface layers. The severe shear deformation induces the grain boundary to be destroyed. With a high enough strain, one can expect the plastic deformation in the sliding testing.

**Figure 14:**
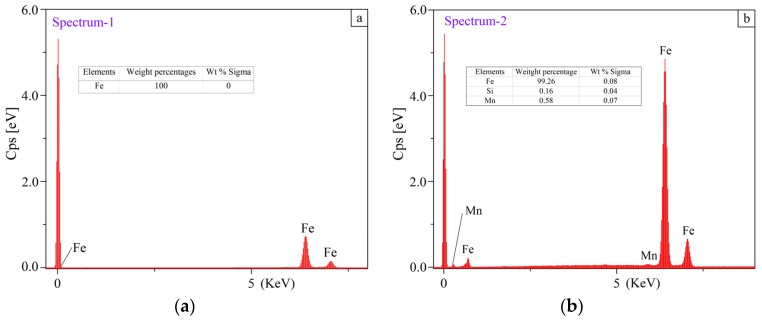

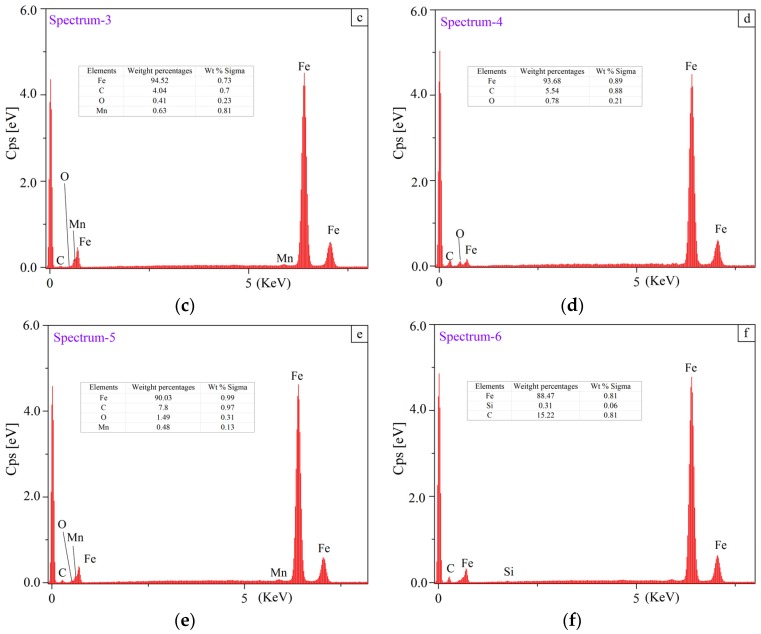
EDS measurement results in the fragmentation zones: (**a**–**f**) EDS spectrums analysis and the elements compositions in the fragmentation zones taken from the squared zones 1–6 marked in Figure 13, respectively.

In this case, the wear debris is trapped and accumulated in the traps of the oscillating surfaces. The rougher the surface is, the stronger the tendency to form debris will be, especially the surface with a high Rku value. The wear particles are generated as the ball ploughs the surface. Detached wear particles experience micro-cutting, adhesive junctions and spalling to form the new surface. Simultaneously, nucleation voids will develop into the cracks on the surface and subsurface layers by further sliding. The aforementioned cracks hasa length of about 10 µm in the subsurface of sample No. 1 (Figure 13a) during the sliding process. Additionally, subsurface cracks will be nucleated, and then the debris and the particles will be generated due to microstructural defects in the material. Generally, the void nucleation and crack development are always generated in the fragmentation zones and the textured grain zone, especially in the former. Depending on the mechanisms of the void nucleation [45], the dislocation pile-up and the grain boundary slip contribute to the void nucleation due to the large plastic strain caused by the sliding. In sliding tests, the higher tangential force leads to a larger plastic strain in the subsurface layer when the ball slides over a rougher surface. Therefore, the voids are liable to be nucleated under a rougher surface in sliding tests. According to the experimental results, it is not difficult to infer that a higher friction coefficient will lead to a thicker plastic deformed layer, which is accompanied by a higher plastic strain under the worn surface. EDS was employed to confirm that the elements change in the fragmentation zone. The results are shown in Figure 14. 

The peaks of ferrite (Fe), carbon (C), silicon (Si), manganese (Mn) and oxygen (O) in the fragmentation zones (marked in Figure 13) are shown together with the EDS spectra (Figure 14). It appears that the chemical compositions are different from each other. The largest weight percentage of Fe is 100%, which was detected in the marked area of specimen No. 1. No other elements were detected except the Fe element. The largest weight percentage of the C element was found in specimen No. 6 after the sliding test, as displayed in Figure 14f. It can be pointed out that a transition was generated in the sliding tests. The results also implied that little cementite was observed and the grains were refined in the fragmentation zone, which was explained with the mechanism of the ultrafine structure in the ductile material [13].

**Figure 15:**
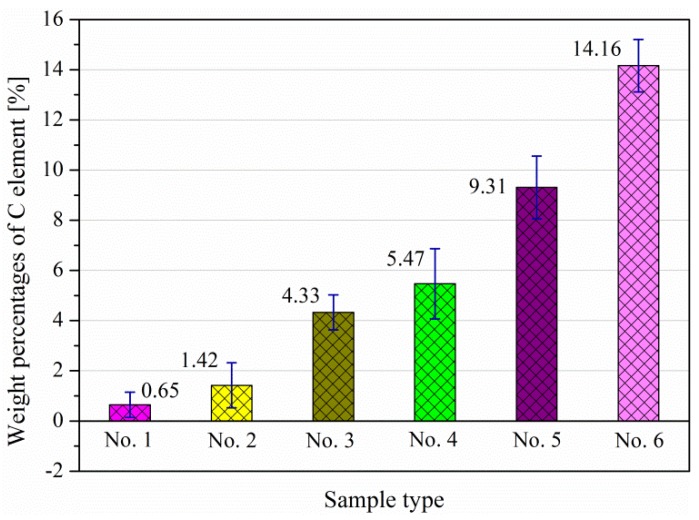
The weight percentages of the C element.

In order to study the C element in the subsurface, EDS measurements performed for the six specimens were derived from three different positions at the same depth of 10 μm under the worn surface. The mean values of the C element content under the worn surfaces are shown in Figure 15.

As presented in the Figure 15, minor weight percentages of C are detected in samples with smaller Ra and Rq values, as well as low Rsk and high Rku values. This implies that the initial roughness parameters affect the loss of the C element. To be confirmed, a surface with high Ra, high Rq, low Rsk and Rku values will generate a larger plastic deformation in a dry sliding test. Such a deformation leads to the abnormal strain misfit dislocation at the grain boundaries under the worn surface layer. The cementite was lost due to the unfavourable performance in the reciprocating extrusion, which is an explanation from another point of view of the voids generated under a rougher surface as well.

## 4. Conclusions

Based on the discussion in the present work, the following five points can be concluded:

(1) AISI 1045 steel surfaces with higher initial roughness values of Ra, Rq and Rku result in a larger average friction coefficient and a longer initial steady wear transition period in the sliding friction tests. Surfaces with low Rsk and high Rku values have little benefit for improving the wear resistance and tribological properties in the dry sliding test.
(2) For the AISI 1045 steel in dry sliding tests, it was found that the weight and the volume loss keep approximately the same level for specimens with initial roughness values below a certain limit (Ra 0.3–0.5 µm, Rq 0.1–0.4 µm). In this case, this is not preferable for improving the wear resistance by further decreasing Ra and Rq.
(3) The plastic deformation mainly concentrates in the depth of 20–50 μm under the worn surface. Critical plastic deformation is generated in the samples with surface roughness parameters of higher Ra, Rq and Rku.
(4) The Fe element weight percentage measured in the fragmentation zones decreases with increasing initial roughness values of Ra and Rq, whereas a 100% content was found in the sample with the lowest initial roughness values of Ra and Rq. This indicates that the refinement of the grains took place during the sliding.
(5) The initial roughness parameters affect the loss of the C element in the worn surface. Surfaces with large Ra, Rq, low Rsk and high Rku values easily lose the C element in dry sliding.

## Figures and Tables

**Table 1:** Chemical compositions of AISI 1045 steel (wt. %).

C	P	Si	Ca	Mn	Mo	Ni	Cr	W	Fe
0.46	0.03	0.31	0.4	0.65	0.08	0.39	0.04	<0.01	Balance

**Table 2:** Detailed information on the roughness and topography of the samples.

Samples	3D Topography of Ground Surfaces	SEM of Ground Surfaces	Ra (μm)	Rq (μm)	Rsk	Rku
No. 1	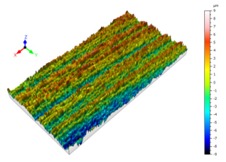	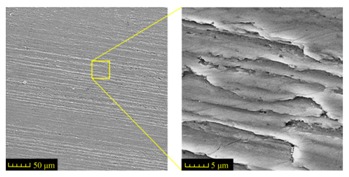	1.127	1.52	−0.182	7.63
No. 2	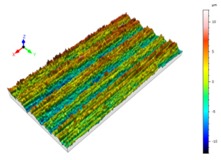	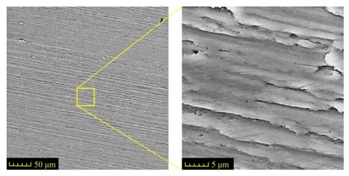	0.805	0.970	−0.0764	3.24
No. 3	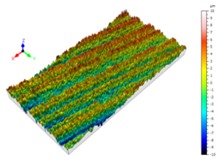	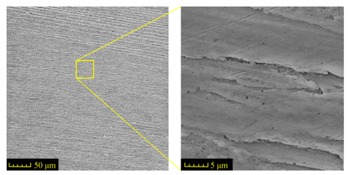	0.689	0.867	0.0748	2.81
No. 4	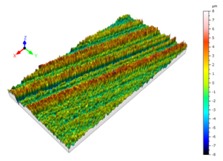	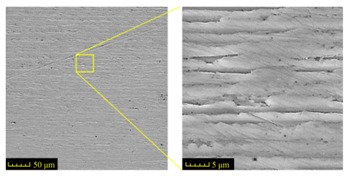	0.449	0.543	0.131	2.70
No. 5	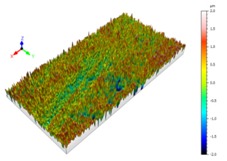	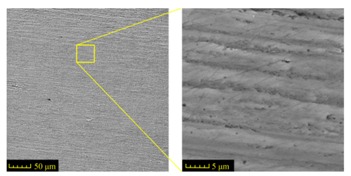	0.263	0.314	0.169	2.44
No. 6	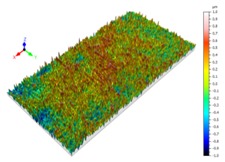	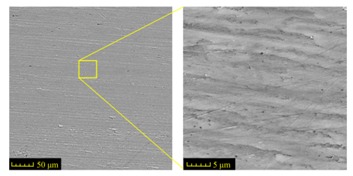	0.108	0.140	0.359	2.13

**Table 3:** Chemical compositions of GGr15 steel (wt %).

C	Si	Mn	Ni	P	S	Mo	Cr	Cu
0.95~1.05	0.15~0.35	0.20~0.40	≤0.03	≤0.027	≤0.02	≤0.10	1.3~1.65	≤0.025
